# 3-Acetyl-2-fluoro-6*H*-benzo[*c*]chromen-6-one

**DOI:** 10.1107/S1600536814005959

**Published:** 2014-03-26

**Authors:** Yoshinobu Ishikawa, Takafumi Suzuki, Nanako Yoshida

**Affiliations:** a School of Pharmaceutical Sciences, University of Shizuoka, 52-1 Yada, Suruga-ku, Shizuoka 422-8526, Japan

## Abstract

The title compound, C_15_H_9_FO_3_, was obtained in a one-pot synthesis by Suzuki–Miyaura cross-coupling and nucleophilic substitution reaction of 4′-chloro-2′,5′-di­fluoro­aceto­phenone with *o*-(meth­oxy­carbon­yl)phenyl­boronic acid. The asymmetric unit contains two crystallographically independent mol­ecules related by a non-crystallographic inversion centre. There are face-to-face stacking inter­actions between the aromatic rings of the benzoate and aceto­phenone units of the symmetry-independent mol­ecules [centroid–centroid distances = 3.870 (3) and 3.986 (3) Å]. In the crystal, mol­ecules are further assembled *via* stacking inter­actions along the *a*-axis direction. One of the mol­ecules inter­acts with its inversion equivalent [centroid–centroid distance between the aromatic rings of the benzoate and aceto­phenone units = 3.932 (3) Å], and the other inter­acts with its twofold axis equivalent [centroid–centroid distance between the aromatic rings of aceto­phenone units = 3.634 (3) Å].

## Related literature   

For background to this study, see: Dias *et al.* (2009 ▶); Ishikawa & Fujii (2011 ▶). For related compounds and structures, see: Bringmann & Menche (2001 ▶); Robinson *et al.* (1991 ▶); Siegel *et al.* (2010 ▶); Dasari *et al.* (2012 ▶). For the biological activity of related compounds, see: Sun *et al.* (2006 ▶).
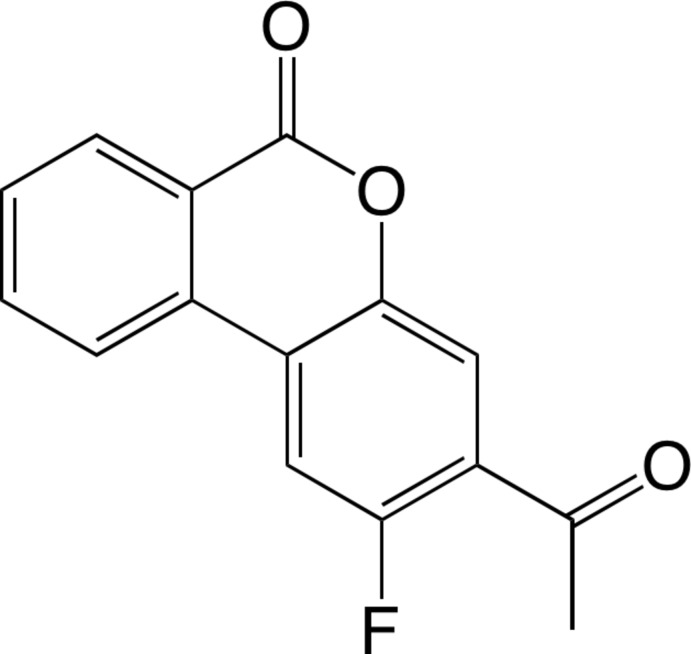


## Experimental   

### 

#### Crystal data  

C_15_H_9_FO_3_
*M* *_r_* = 256.23
Monoclinic, 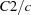

*a* = 26.005 (19) Å
*b* = 13.169 (4) Å
*c* = 13.297 (8) Å
β = 98.02 (6)°
*V* = 4509 (5) Å^3^
*Z* = 16
Mo *K*α radiation
μ = 0.12 mm^−1^
*T* = 100 K
0.40 × 0.34 × 0.25 mm

#### Data collection  

Rigaku AFC-7R diffractometer
6119 measured reflections
5160 independent reflections
3534 reflections with *F* ^2^ > 2σ(*F* ^2^)
*R*_int_ = 0.017
3 standard reflections every 150 reflections intensity decay: −0.4%

#### Refinement  

*R*[*F* ^2^ > 2σ(*F* ^2^)] = 0.047
*wR*(*F* ^2^) = 0.137
*S* = 1.01
5160 reflections
343 parameters
H-atom parameters constrained
Δρ_max_ = 0.35 e Å^−3^
Δρ_min_ = −0.28 e Å^−3^

### 

Data collection: *WinAFC Diffractometer Control Software* (Rigaku, 1999 ▶); cell refinement: *WinAFC Diffractometer Control Software*; data reduction: *WinAFC Diffractometer Control Software*; program(s) used to solve structure: *SIR2008* (Burla *et al.*, 2007 ▶); program(s) used to refine structure: *SHELXL97* (Sheldrick, 2008 ▶); molecular graphics: *CrystalStructure* (Rigaku, 2010 ▶); software used to prepare material for publication: *CrystalStructure*.

## Supplementary Material

Crystal structure: contains datablock(s) General, I. DOI: 10.1107/S1600536814005959/fy2113sup1.cif

Structure factors: contains datablock(s) I. DOI: 10.1107/S1600536814005959/fy2113Isup2.hkl

Supporting information file. DOI: 10.1107/S1600536814005959/fy2113Isup3.cml

CCDC reference: 992229

Additional supporting information:  crystallographic information; 3D view; checkCIF report

## Figures and Tables

**Fig. 1.**
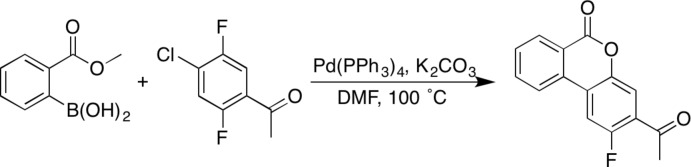
Reaction scheme for the title compound.

**Fig. 2.**
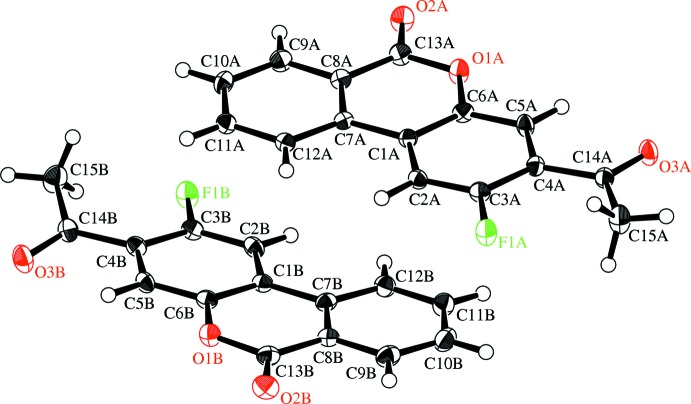
The molecular structure of the title compound, with displacement ellipsoids drawn at the 50% probability level. Hydrogen atoms are shown as small spheres of arbitrary radius.

## supplementary crystallographic information

### 1. Comment

Aryl diketo acids are known to inhibit influenza virus metalloenzyme endonuclease by chelating its metal center (Dias *et al.*,2009). According to our inhibitor design targeting this metalloenzyme (Ishikawa & Fujii, 2011), we tried to synthesize a biphenyl derivative with acetyl, methoxycarbonyl and difluoro groups by Suzuki-Miyaura cross-coupling reaction of 4'-chloro-2',5'-difluoroacetophenone with *o*-(methoxycarbonyl)phenylboronic acid in *N*,*N*-dimethylformamide (DMF) in the presence of Pd(PPh_3_)_4_ and K_2_CO_3_. Usual work-up yielded a pink solid, which was not fully analyzed by ^1^H NMR and MS.

The crystallographic analysis revealed that it is an unexpected biphenyl lactone derivative shown in Fig.1, which should be formed as a result of intramolecular cyclization by additional nucleophilic substitution reaction of the hydrolyzed carboxylate with the proximal fluoride group after the intermolecular cross-coupling reaction. This result well accounts for the ^1^H NMR and MS spectra. The asymmetric unit of the title compound contains two crystallographically independent molecules related by a non-crystallographic inversion centre. The biphenyl lactone rings are essentially planar, and the mean atomic deviations from the corresponding least-square planes composed of the non-hydrogen atoms of the rings are 0.0137 and 0.0188 Å for molecules A and B, respectively. In addition, face-to-face stacking interactions between the aromatic rings of the benzoate and acetophenone units are observed [centroid–centroid distances = 3.870 (3) and 3.986 (3) Å]. In the crystal, the molecules are further assembled *via* stacking interaction along the *a*-axis direction. Molecule A interacts with its inversion equivalent [centroid-centroid distance between the aromatic rings of the benzoate and acetophenone units = 3.932 (3) Å], and molecule B interacts with its twofold axis equivalent [centroid-centroid distance between the aromatic rings of acetophenone units = 3.634 (3) Å].

Biphenyl lactone is a key structural motif found in many natural products and pharmaceuticals (Bringmann *et al.*, 2001). The crystal structures (Robinson *et al.*,1991; Siegel *et al.*, 2010, Dasari *et al.*, 2012) and biological activity (Sun *et al.*, 2006) of the related compounds are reported.

### 2. Experimental

In a Schlenk tube under nitrogen atmosphere, the mixture of 4'-chloro-2',5'-difluoroacetophenone (1.0 g, 0.0052 mol), *o*-(methoxycarbonyl)phenylboronic acid (0.83 g, 0.0046 mol), K_2_CO_3_ (0.87 g, 0.0063 mol) and Pd(PPh_3_)_4_ (0.15 g, 0.00013 mol) in 30 ml of DMF were stirred at 100 °C for 18 h. After cooling to room temperature and filtration, ethyl acetate and water were added to the filtrate. The organic phase was separated, washed with water and brine, dried with MgSO_4_, and was slowly evaporated to give the pink solid (yield: 14%). ^1^H NMR (400 MHz, CDCl_3_): *d* = 2.71 (d, 3H, *J* = 4.8 Hz), 7.71 (t, 1H, *J* = 8.0 Hz), 7.82 (d, 1H, *J* = 11.2 Hz), 7.88 (t, 1H, *J* = 8.0 Hz), 7.91 (d, 1H, *J* = 8.0 Hz), 7.08 (d, 1H, *J* = 8.0 Hz), 8.46 (d, 1H, *J* = 8.0 Hz). DART-MS calcd for [C_15_H_9_FO_3_ + H^+^]: 257.061, found 257.081. Single crystals suitable for X-ray diffraction were obtained by recrystalization of an ethyl acetate solution of the title compound at room temperature.

### 3. Refinement

The hydrogen atoms were placed in geometrical positions [C–H 0.95 Å for phenyl, C–H 0.98 Å for methyl, *U*_iso_(H) = 1.2*U*_eq_(C)], and refined using a riding model.

### Crystal data

**Table:**

C_15_H_9_FO_3_	*F*(000) = 2112.00
*M**_r_* = 256.23	*D*_x_ = 1.510 Mg m^−^^3^
Monoclinic, *C*2/*c*	Mo *K*α radiation, λ = 0.71069 Å
Hall symbol: -C 2yc	Cell parameters from 25 reflections
*a* = 26.005 (19) Å	θ = 15.6–17.4°
*b* = 13.169 (4) Å	µ = 0.12 mm^−^^1^
*c* = 13.297 (8) Å	*T* = 100 K
β = 98.02 (6)°	Block, pink
*V* = 4509 (5) Å^3^	0.40 × 0.34 × 0.25 mm
*Z* = 16	

### Data collection

**Table:**

Rigaku AFC-7R diffractometer	θ_max_ = 27.5°
ω scans	*h* = −18→33
6119 measured reflections	*k* = 0→17
5160 independent reflections	*l* = −17→16
3534 reflections with *F*^2^ > 2σ(*F*^2^)	3 standard reflections every 150 reflections
*R*_int_ = 0.017	intensity decay: −0.4%

### Refinement

**Table:**

Refinement on *F*^2^	Secondary atom site location: difference Fourier map
*R*[*F*^2^ > 2σ(*F*^2^)] = 0.047	Hydrogen site location: inferred from neighbouring sites
*wR*(*F*^2^) = 0.137	H-atom parameters constrained
*S* = 1.01	*w* = 1/[σ^2^(*F*_o_^2^) + (0.0742*P*)^2^ + 2.0957*P*] where *P* = (*F*_o_^2^ + 2*F*_c_^2^)/3
5160 reflections	(Δ/σ)_max_ = 0.001
343 parameters	Δρ_max_ = 0.35 e Å^−^^3^
0 restraints	Δρ_min_ = −0.28 e Å^−^^3^
Primary atom site location: structure-invariant direct methods	

### Special details

**Table:**

Refinement. Refinement was performed using all reflections. The weighted *R*-factor (*wR*) and goodness of fit (*S*) are based on *F*^2^. *R*-factor (gt) are based on *F*. The threshold expression of *F*^2^ > 2.0 σ(*F*^2^) is used only for calculating *R*-factor (gt).

### Fractional atomic coordinates and isotropic or equivalent isotropic displacement parameters (Å^2^)

**Table:**

	*x*	*y*	*z*	*U*_iso_*/*U*_eq_	
F1A	0.31263 (5)	1.07390 (8)	0.04611 (8)	0.0259 (3)	
F1B	0.44773 (4)	0.42794 (8)	0.02258 (8)	0.0251 (3)	
O1A	0.31217 (5)	0.78443 (9)	−0.24820 (9)	0.0216 (3)	
O2A	0.31393 (5)	0.62794 (10)	−0.30292 (10)	0.0246 (3)	
O3A	0.30739 (5)	1.15534 (10)	−0.25742 (9)	0.0234 (3)	
O1B	0.42805 (5)	0.71154 (10)	0.31463 (9)	0.0202 (3)	
O2B	0.42512 (5)	0.86817 (10)	0.37147 (10)	0.0258 (3)	
O3B	0.44045 (6)	0.33924 (11)	0.31809 (10)	0.0277 (4)	
C1A	0.31365 (6)	0.82575 (13)	−0.06925 (12)	0.0150 (4)	
C2A	0.31386 (7)	0.90307 (13)	0.00280 (13)	0.0171 (4)	
C3A	0.31233 (7)	1.00254 (14)	−0.02744 (13)	0.0175 (4)	
C4A	0.31038 (7)	1.03261 (13)	−0.12886 (13)	0.0167 (4)	
C5A	0.31047 (7)	0.95538 (13)	−0.20015 (13)	0.0181 (4)	
C6A	0.31227 (7)	0.85494 (13)	−0.17052 (13)	0.0171 (4)	
C7A	0.31431 (6)	0.71708 (13)	−0.04513 (13)	0.0152 (4)	
C8A	0.31428 (7)	0.64746 (14)	−0.12495 (13)	0.0175 (4)	
C9A	0.31458 (7)	0.54330 (14)	−0.10615 (14)	0.0217 (4)	
C10A	0.31479 (7)	0.50751 (14)	−0.00851 (14)	0.0207 (4)	
C11A	0.31461 (7)	0.57649 (14)	0.07141 (14)	0.0195 (4)	
C13A	0.31366 (7)	0.68230 (14)	−0.23014 (14)	0.0201 (4)	
C14A	0.30814 (7)	1.14026 (13)	−0.16662 (13)	0.0176 (4)	
C15A	0.30670 (9)	1.22769 (15)	−0.09546 (14)	0.0283 (5)	
C1B	0.44025 (6)	0.67388 (14)	0.14047 (13)	0.0165 (4)	
C2B	0.44531 (7)	0.59797 (14)	0.06830 (13)	0.0186 (4)	
C3B	0.44303 (7)	0.49787 (14)	0.09551 (13)	0.0175 (4)	
C4B	0.43642 (7)	0.46580 (14)	0.19314 (13)	0.0174 (4)	
C5B	0.43176 (7)	0.54154 (14)	0.26455 (13)	0.0176 (4)	
C6B	0.43337 (7)	0.64274 (14)	0.23804 (13)	0.0168 (4)	
C14B	0.43523 (7)	0.35764 (14)	0.22744 (14)	0.0199 (4)	
C7B	0.44059 (6)	0.78242 (14)	0.11788 (13)	0.0176 (4)	
C12B	0.44570 (7)	0.82098 (15)	0.02167 (14)	0.0205 (4)	
C11B	0.44476 (7)	0.92443 (15)	0.00505 (15)	0.0235 (4)	
C10B	0.43892 (8)	0.99194 (15)	0.08315 (16)	0.0252 (5)	
C9B	0.43407 (7)	0.95588 (15)	0.17896 (15)	0.0234 (4)	
C12A	0.31442 (6)	0.67979 (14)	0.05381 (13)	0.0170 (4)	
C13B	0.42922 (7)	0.81498 (14)	0.29949 (14)	0.0203 (4)	
C15B	0.42691 (9)	0.27294 (15)	0.15133 (15)	0.0289 (5)	
C8B	0.43480 (7)	0.85080 (14)	0.19627 (13)	0.0177 (4)	
H1A	0.3151	0.8866	0.0727	0.0205*	
H2A	0.3093	0.9720	−0.2700	0.0217*	
H3A	0.3146	0.4968	−0.1607	0.0260*	
H4A	0.3151	0.4365	0.0043	0.0248*	
H5A	0.3146	0.5520	0.1386	0.0234*	
H6A	0.3144	0.7258	0.1088	0.0204*	
H7A	0.2788	1.2171	−0.0539	0.0339*	
H8A	0.3003	1.2906	−0.1345	0.0339*	
H9A	0.3401	1.2327	−0.0512	0.0339*	
H1B	0.4503	0.6159	0.0011	0.0223*	
H2B	0.4275	0.5234	0.3320	0.0211*	
H6B	0.4498	0.7758	−0.0323	0.0246*	
H5B	0.4481	0.9499	−0.0606	0.0282*	
H4B	0.4383	1.0630	0.0706	0.0302*	
H3B	0.4303	1.0018	0.2326	0.0280*	
H7B	0.4272	0.2078	0.1870	0.0346*	
H8B	0.4547	0.2738	0.1086	0.0346*	
H9B	0.3933	0.2820	0.1087	0.0346*	

### Atomic displacement parameters (Å^2^)

**Table:**

	*U*^11^	*U*^22^	*U*^33^	*U*^12^	*U*^13^	*U*^23^
F1A	0.0483 (7)	0.0158 (6)	0.0137 (6)	−0.0002 (5)	0.0051 (5)	−0.0042 (5)
F1B	0.0400 (7)	0.0202 (6)	0.0162 (6)	0.0028 (5)	0.0074 (5)	−0.0029 (5)
O1A	0.0361 (8)	0.0167 (7)	0.0129 (6)	−0.0005 (6)	0.0073 (6)	−0.0025 (5)
O2A	0.0370 (8)	0.0195 (7)	0.0184 (7)	0.0000 (6)	0.0079 (6)	−0.0055 (6)
O3A	0.0351 (8)	0.0206 (7)	0.0148 (6)	−0.0002 (6)	0.0048 (6)	0.0026 (6)
O1B	0.0272 (7)	0.0195 (7)	0.0146 (6)	0.0014 (6)	0.0049 (5)	−0.0027 (5)
O2B	0.0309 (8)	0.0260 (8)	0.0206 (7)	0.0018 (6)	0.0041 (6)	−0.0072 (6)
O3B	0.0417 (9)	0.0235 (8)	0.0177 (7)	0.0027 (7)	0.0032 (6)	0.0033 (6)
C1A	0.0149 (8)	0.0161 (9)	0.0141 (8)	−0.0004 (7)	0.0027 (6)	−0.0002 (7)
C2A	0.0211 (9)	0.0177 (9)	0.0122 (8)	−0.0010 (7)	0.0017 (7)	−0.0006 (7)
C3A	0.0228 (9)	0.0165 (9)	0.0134 (8)	−0.0001 (7)	0.0027 (7)	−0.0040 (7)
C4A	0.0172 (9)	0.0163 (9)	0.0169 (9)	−0.0003 (7)	0.0035 (7)	0.0017 (7)
C5A	0.0244 (9)	0.0178 (9)	0.0125 (8)	0.0005 (7)	0.0047 (7)	0.0021 (7)
C6A	0.0204 (9)	0.0165 (9)	0.0149 (8)	−0.0009 (7)	0.0043 (7)	−0.0050 (7)
C7A	0.0142 (8)	0.0146 (9)	0.0167 (8)	−0.0006 (7)	0.0022 (7)	−0.0013 (7)
C8A	0.0173 (9)	0.0184 (9)	0.0172 (9)	−0.0001 (7)	0.0038 (7)	−0.0010 (7)
C9A	0.0244 (10)	0.0188 (10)	0.0218 (9)	0.0001 (8)	0.0031 (8)	−0.0025 (8)
C10A	0.0236 (9)	0.0138 (9)	0.0247 (10)	−0.0001 (7)	0.0040 (8)	0.0010 (8)
C11A	0.0172 (9)	0.0206 (10)	0.0203 (9)	−0.0003 (7)	0.0013 (7)	0.0028 (7)
C13A	0.0227 (9)	0.0190 (9)	0.0188 (9)	0.0014 (8)	0.0039 (7)	−0.0028 (8)
C14A	0.0198 (9)	0.0158 (9)	0.0171 (9)	−0.0001 (7)	0.0024 (7)	0.0010 (7)
C15A	0.0486 (13)	0.0186 (10)	0.0184 (10)	0.0013 (9)	0.0075 (9)	−0.0017 (8)
C1B	0.0143 (8)	0.0193 (9)	0.0157 (8)	0.0001 (7)	0.0017 (7)	−0.0009 (7)
C2B	0.0190 (9)	0.0235 (10)	0.0134 (8)	0.0017 (7)	0.0029 (7)	0.0013 (7)
C3B	0.0196 (9)	0.0194 (10)	0.0140 (8)	0.0019 (7)	0.0035 (7)	−0.0042 (7)
C4B	0.0184 (9)	0.0188 (10)	0.0150 (8)	0.0017 (7)	0.0021 (7)	−0.0010 (7)
C5B	0.0200 (9)	0.0211 (10)	0.0118 (8)	0.0005 (7)	0.0031 (7)	0.0017 (7)
C6B	0.0165 (8)	0.0195 (9)	0.0145 (8)	0.0007 (7)	0.0022 (7)	−0.0026 (7)
C14B	0.0214 (9)	0.0200 (10)	0.0189 (9)	0.0027 (8)	0.0047 (7)	0.0015 (8)
C7B	0.0134 (8)	0.0179 (9)	0.0213 (9)	−0.0007 (7)	0.0016 (7)	−0.0010 (7)
C12B	0.0195 (9)	0.0233 (10)	0.0190 (9)	−0.0009 (8)	0.0036 (7)	0.0013 (8)
C11B	0.0231 (10)	0.0233 (10)	0.0244 (10)	−0.0015 (8)	0.0038 (8)	0.0029 (8)
C10B	0.0241 (10)	0.0184 (10)	0.0331 (11)	−0.0025 (8)	0.0037 (8)	0.0021 (8)
C9B	0.0227 (10)	0.0188 (10)	0.0282 (10)	−0.0007 (8)	0.0023 (8)	−0.0042 (8)
C12A	0.0161 (8)	0.0181 (9)	0.0165 (9)	0.0002 (7)	0.0014 (7)	−0.0000 (7)
C13B	0.0191 (9)	0.0203 (10)	0.0209 (9)	−0.0001 (7)	0.0010 (7)	−0.0037 (8)
C15B	0.0417 (12)	0.0212 (11)	0.0248 (10)	−0.0034 (9)	0.0085 (9)	−0.0041 (8)
C8B	0.0156 (8)	0.0196 (10)	0.0177 (9)	−0.0010 (7)	0.0010 (7)	−0.0016 (7)

### Geometric parameters (Å, º)

**Table:**

F1A—C3A	1.356 (3)	C3B—C4B	1.398 (3)
F1B—C3B	1.355 (3)	C4B—C5B	1.394 (3)
O1A—C6A	1.389 (3)	C4B—C14B	1.497 (3)
O1A—C13A	1.366 (3)	C5B—C6B	1.381 (3)
O2A—C13A	1.205 (3)	C14B—C15B	1.501 (3)
O3A—C14A	1.221 (3)	C7B—C12B	1.400 (3)
O1B—C6B	1.384 (3)	C7B—C8B	1.401 (3)
O1B—C13B	1.378 (3)	C12B—C11B	1.380 (3)
O2B—C13B	1.203 (3)	C11B—C10B	1.391 (3)
O3B—C14B	1.218 (3)	C10B—C9B	1.382 (3)
C1A—C2A	1.398 (3)	C9B—C8B	1.403 (3)
C1A—C6A	1.396 (3)	C13B—C8B	1.478 (3)
C1A—C7A	1.466 (3)	C2A—H1A	0.950
C2A—C3A	1.369 (3)	C5A—H2A	0.950
C3A—C4A	1.400 (3)	C9A—H3A	0.950
C4A—C5A	1.391 (3)	C10A—H4A	0.950
C4A—C14A	1.502 (3)	C11A—H5A	0.950
C5A—C6A	1.379 (3)	C15A—H7A	0.980
C7A—C8A	1.402 (3)	C15A—H8A	0.980
C7A—C12A	1.404 (3)	C15A—H9A	0.980
C8A—C9A	1.394 (3)	C2B—H1B	0.950
C8A—C13A	1.470 (3)	C5B—H2B	0.950
C9A—C10A	1.381 (3)	C12B—H6B	0.950
C10A—C11A	1.399 (3)	C11B—H5B	0.950
C11A—C12A	1.380 (3)	C10B—H4B	0.950
C14A—C15A	1.494 (3)	C9B—H3B	0.950
C1B—C2B	1.405 (3)	C12A—H6A	0.950
C1B—C6B	1.396 (3)	C15B—H7B	0.980
C1B—C7B	1.461 (3)	C15B—H8B	0.980
C2B—C3B	1.370 (3)	C15B—H9B	0.980
			
F1A···C14A	2.947 (3)	C7B···H3B	3.2949
F1A···C15A	2.755 (3)	C12B···H1B	2.7190
F1B···C14B	2.938 (3)	C12B···H4B	3.2637
F1B···C15B	2.766 (3)	C11B···H3B	3.2631
O1A···C7A	2.835 (3)	C10B···H6B	3.2652
O2A···C6A	3.473 (3)	C9B···H5B	3.2572
O2A···C9A	2.842 (3)	C12A···H1A	2.7352
O3A···C5A	2.739 (3)	C12A···H4A	3.2710
O1B···C7B	2.839 (3)	C13B···H3B	2.6177
O2B···C6B	3.480 (3)	C8B···H6B	3.2710
O2B···C9B	2.847 (3)	C8B···H4B	3.2636
O3B···C5B	2.759 (3)	H1A···H6A	2.1726
C1A···C4A	2.835 (3)	H3A···H4A	2.3321
C1A···C13A	2.854 (3)	H4A···H5A	2.3464
C2A···C5A	2.775 (3)	H5A···H6A	2.3231
C2A···C12A	3.017 (3)	H1B···H6B	2.1521
C3A···C6A	2.720 (3)	H6B···H5B	2.3225
C3A···C15A	3.097 (3)	H5B···H4B	2.3346
C6A···C8A	2.798 (3)	H4B···H3B	2.3356
C7A···C10A	2.802 (3)	F1A···H2A^i^	2.5325
C8A···C11A	2.772 (3)	F1A···H4A^ii^	3.2972
C9A···C12A	2.785 (3)	F1A···H4B	3.2409
C1B···C4B	2.834 (3)	F1A···H9B^xi^	3.4807
C1B···C13B	2.859 (3)	F1B···H4A	3.4268
C2B···C5B	2.783 (3)	F1B···H1B^iii^	2.7751
C2B···C12B	3.002 (3)	F1B···H2B^iv^	2.5957
C3B···C6B	2.725 (3)	O1A···H7A^vii^	3.4018
C3B···C15B	3.096 (3)	O1A···H8A^vii^	3.1165
C6B···C8B	2.797 (3)	O1A···H7B^iv^	3.2290
C7B···C10B	2.797 (3)	O1A···H9B^iv^	3.1562
C12B···C9B	2.793 (3)	O2A···H4A^iv^	2.7044
C11B···C8B	2.767 (3)	O2A···H5A^iv^	2.4943
F1A···O1A^i^	3.312 (2)	O2A···H7A^vii^	3.0891
F1A···C5A^i^	3.404 (3)	O2A···H9B^iv^	2.7803
F1A···C10A^ii^	3.452 (4)	O3A···H1A^vi^	2.3615
F1A···C10B	3.427 (4)	O3A···H6A^vi^	2.3950
F1B···F1B^iii^	3.436 (3)	O1B···H8A^i^	3.4828
F1B···O1B^iv^	3.300 (2)	O1B···H9A^i^	3.1774
F1B···O3B^v^	3.548 (3)	O2B···H9A^i^	2.8892
F1B···C10A	3.581 (4)	O2B···H5B^i^	2.6011
F1B···C2B^iii^	3.202 (3)	O2B···H4B^i^	2.7742
F1B···C3B^iii^	3.569 (3)	O3B···H1B^ix^	2.4828
F1B···C5B^iv^	3.422 (3)	O3B···H6B^ix^	2.4851
O1A···F1A^vi^	3.312 (2)	O3B···H8B^v^	2.8952
O1A···O3A^vii^	3.553 (3)	C1A···H6A^ii^	3.3663
O1A···C15A^vii^	3.556 (4)	C1A···H6B	3.5681
O1A···C15B^iv^	3.517 (4)	C2A···H2A^i^	3.4573
O2A···O3A^vii^	3.381 (3)	C3A···H2A^i^	3.4521
O2A···O3B^iv^	3.482 (3)	C3A···H4A^ii^	3.4635
O2A···C4A^vii^	3.469 (3)	C3A···H5A^ii^	3.4979
O2A···C10A^iv^	3.267 (3)	C3A···H4B	3.4446
O2A···C11A^iv^	3.170 (3)	C4A···H5A^ii^	3.4212
O2A···C14A^vii^	3.148 (4)	C5A···H3A^viii^	3.5531
O2A···C15A^vii^	3.494 (4)	C5A···H5A^ii^	3.4644
O2A···C4B^iv^	3.423 (3)	C5A···H3B^vi^	3.4070
O2A···C14B^iv^	3.130 (4)	C7A···H6A^ii^	3.4191
O2A···C15B^iv^	3.347 (4)	C7A···H6B	3.5890
O3A···O1A^viii^	3.553 (3)	C8A···H1A^ii^	3.5566
O3A···O2A^viii^	3.381 (3)	C9A···H1A^ii^	3.5823
O3A···O2B^vi^	3.308 (3)	C9A···H2A^vii^	3.5341
O3A···C2A^vi^	3.307 (3)	C9A···H8A^xii^	3.3639
O3A···C8A^viii^	3.330 (4)	C9A···H2B^iv^	3.2766
O3A···C13A^viii^	3.149 (4)	C10A···H8A^xii^	3.3066
O3A···C12A^vi^	3.343 (3)	C13A···H5A^iv^	3.5463
O3A···C13B^vi^	3.177 (4)	C13A···H7A^vii^	3.5091
O3A···C8B^vi^	3.454 (4)	C13A···H8A^vii^	3.5439
O1B···F1B^ix^	3.300 (2)	C13A···H7B^iv^	3.5973
O1B···C1B^v^	3.429 (4)	C13A···H9B^iv^	3.2166
O1B···C7B^v^	3.534 (3)	C14A···H1A^vi^	3.5145
O2B···O3A^i^	3.308 (3)	C14A···H6A^vi^	3.4911
O2B···C4A^i^	3.257 (3)	C15A···H4A^xi^	3.0480
O2B···C14A^i^	3.015 (3)	C15A···H7A^xiii^	3.2645
O2B···C15A^i^	3.413 (4)	C15A···H9B^xi^	3.3534
O2B···C12B^v^	3.515 (4)	C1B···H6A	3.3130
O2B···C11B^i^	3.259 (3)	C2B···H2B^v^	3.5280
O2B···C10B^i^	3.341 (3)	C2B···H2B^iv^	3.4978
O3B···F1B^v^	3.548 (3)	C3B···H4A	3.4747
O3B···O2A^ix^	3.482 (3)	C3B···H5A	3.5387
O3B···C8A^ix^	3.473 (4)	C3B···H1B^iii^	3.5481
O3B···C13A^ix^	3.282 (4)	C3B···H2B^v^	3.3883
O3B···C2B^ix^	3.413 (3)	C3B···H2B^iv^	3.4818
O3B···C14B^v^	3.380 (4)	C4B···H5A	3.3476
O3B···C12B^ix^	3.420 (3)	C5B···H3A^ix^	3.3708
O3B···C15B^v^	3.526 (4)	C5B···H5A	3.2682
C1A···C11A^ii^	3.572 (4)	C6B···H5A	3.4023
C1A···C12B	3.478 (4)	C6B···H6A	3.4987
C1A···C12A^ii^	3.366 (4)	C14B···H8B^v^	3.5242
C2A···O3A^i^	3.307 (3)	C7B···H1A	3.5150
C2A···C10A^ii^	3.558 (4)	C7B···H6A	3.3517
C2A···C11A^ii^	3.360 (4)	C12B···H8B^iii^	3.5381
C2A···C12B	3.570 (4)	C11B···H5B^x^	3.2354
C2A···C11B	3.412 (4)	C11B···H4B^x^	3.3387
C2A···C10B	3.481 (4)	C10B···H1A	3.4919
C2A···C12A^ii^	3.491 (4)	C10B···H5B^x^	3.0902
C3A···C10A^ii^	3.408 (4)	C10B···H7B^xi^	3.1932
C3A···C11A^ii^	3.433 (4)	C9B···H1A	3.3449
C3A···C11B	3.562 (4)	C9B···H2A^i^	3.5370
C3A···C10B	3.416 (4)	C9B···H7B^xi^	3.3247
C4A···O2A^viii^	3.469 (3)	C13B···H9A^i^	3.3158
C4A···O2B^vi^	3.257 (3)	C15B···H9A^xii^	3.3075
C5A···F1A^vi^	3.404 (3)	C15B···H4B^xii^	2.9956
C7A···C12A^ii^	3.600 (4)	C8B···H1A	3.3457
C8A···O3A^vii^	3.330 (4)	C8B···H6A	3.5852
C8A···O3B^iv^	3.473 (4)	H1A···O3A^i^	2.3615
C10A···F1A^ii^	3.452 (4)	H1A···C8A^ii^	3.5566
C10A···F1B	3.581 (4)	H1A···C9A^ii^	3.5823
C10A···O2A^ix^	3.267 (3)	H1A···C14A^i^	3.5145
C10A···C2A^ii^	3.558 (4)	H1A···C7B	3.5150
C10A···C3A^ii^	3.408 (4)	H1A···C10B	3.4919
C10A···C3B	3.430 (4)	H1A···C9B	3.3449
C11A···O2A^ix^	3.170 (3)	H1A···C8B	3.3457
C11A···C1A^ii^	3.572 (4)	H1A···H2A^i^	2.8209
C11A···C2A^ii^	3.360 (4)	H2A···F1A^vi^	2.5325
C11A···C3A^ii^	3.433 (4)	H2A···C2A^vi^	3.4573
C11A···C1B	3.511 (4)	H2A···C3A^vi^	3.4521
C11A···C2B	3.417 (4)	H2A···C9A^viii^	3.5341
C11A···C3B	3.468 (4)	H2A···C9B^vi^	3.5370
C13A···O3A^vii^	3.149 (4)	H2A···H1A^vi^	2.8209
C13A···O3B^iv^	3.282 (4)	H2A···H3A^viii^	3.2422
C13A···C14A^vii^	3.318 (4)	H2A···H3B^vi^	3.1616
C13A···C14B^iv^	3.330 (4)	H3A···C5A^vii^	3.5531
C13A···C15B^iv^	3.578 (4)	H3A···C5B^iv^	3.3708
C14A···O2A^viii^	3.148 (4)	H3A···H2A^vii^	3.2422
C14A···O2B^vi^	3.015 (3)	H3A···H5A^iv^	2.7443
C14A···C13A^viii^	3.318 (4)	H3A···H8A^xii^	2.7692
C14A···C13B^vi^	3.296 (4)	H3A···H2B^iv^	2.9605
C15A···O1A^viii^	3.556 (4)	H4A···F1A^ii^	3.2972
C15A···O2A^viii^	3.494 (4)	H4A···F1B	3.4268
C15A···O2B^vi^	3.413 (4)	H4A···O2A^ix^	2.7044
C1B···O1B^v^	3.429 (4)	H4A···C3A^ii^	3.4635
C1B···C11A	3.511 (4)	H4A···C15A^xii^	3.0480
C1B···C6B^v^	3.478 (4)	H4A···C3B	3.4747
C1B···C12A	3.315 (4)	H4A···H7A^xii^	3.1032
C2B···F1B^iii^	3.202 (3)	H4A···H7A^ii^	3.3081
C2B···O3B^iv^	3.413 (3)	H4A···H8A^xii^	2.6554
C2B···C11A	3.417 (4)	H4A···H9A^xii^	2.8826
C2B···C12A	3.550 (4)	H4A···H9B	3.0698
C3B···F1B^iii^	3.569 (3)	H5A···O2A^ix^	2.4943
C3B···C10A	3.430 (4)	H5A···C3A^ii^	3.4979
C3B···C11A	3.468 (4)	H5A···C4A^ii^	3.4212
C3B···C5B^v^	3.564 (4)	H5A···C5A^ii^	3.4644
C4B···O2A^ix^	3.423 (3)	H5A···C13A^ix^	3.5463
C4B···C4B^v^	3.439 (4)	H5A···C3B	3.5387
C4B···C5B^v^	3.539 (4)	H5A···C4B	3.3476
C5B···F1B^ix^	3.422 (3)	H5A···C5B	3.2682
C5B···C3B^v^	3.564 (4)	H5A···C6B	3.4023
C5B···C4B^v^	3.539 (4)	H5A···H3A^ix^	2.7443
C6B···C1B^v^	3.478 (4)	H6A···O3A^i^	2.3950
C6B···C6B^v^	3.436 (4)	H6A···C1A^ii^	3.3663
C14B···O2A^ix^	3.130 (4)	H6A···C7A^ii^	3.4191
C14B···O3B^v^	3.380 (4)	H6A···C14A^i^	3.4911
C14B···C13A^ix^	3.330 (4)	H6A···C1B	3.3130
C14B···C14B^v^	3.338 (4)	H6A···C6B	3.4987
C7B···O1B^v^	3.534 (3)	H6A···C7B	3.3517
C7B···C12A	3.541 (3)	H6A···C8B	3.5852
C7B···C13B^v^	3.437 (4)	H6A···H8A^i^	3.4894
C12B···O2B^v^	3.515 (4)	H7A···O1A^viii^	3.4018
C12B···O3B^iv^	3.420 (3)	H7A···O2A^viii^	3.0891
C12B···C1A	3.478 (4)	H7A···C13A^viii^	3.5091
C12B···C2A	3.570 (4)	H7A···C15A^xiii^	3.2645
C11B···O2B^vi^	3.259 (3)	H7A···H4A^xi^	3.1032
C11B···C2A	3.412 (4)	H7A···H4A^ii^	3.3081
C11B···C3A	3.562 (4)	H7A···H7A^xiii^	2.3789
C11B···C11B^x^	3.513 (4)	H7A···H8A^xiii^	3.4586
C11B···C10B^x^	3.568 (4)	H7A···H9B^xi^	3.5311
C10B···F1A	3.427 (4)	H8A···O1A^viii^	3.1165
C10B···O2B^vi^	3.341 (3)	H8A···O1B^vi^	3.4828
C10B···C2A	3.481 (4)	H8A···C9A^xi^	3.3639
C10B···C3A	3.416 (4)	H8A···C10A^xi^	3.3066
C10B···C11B^x^	3.568 (4)	H8A···C13A^viii^	3.5439
C12A···O3A^i^	3.343 (3)	H8A···H3A^xi^	2.7692
C12A···C1A^ii^	3.366 (4)	H8A···H4A^xi^	2.6554
C12A···C2A^ii^	3.491 (4)	H8A···H6A^vi^	3.4894
C12A···C7A^ii^	3.600 (4)	H8A···H7A^xiii^	3.4586
C12A···C1B	3.315 (4)	H9A···O1B^vi^	3.1774
C12A···C2B	3.550 (4)	H9A···O2B^vi^	2.8892
C12A···C7B	3.541 (3)	H9A···C13B^vi^	3.3158
C13B···O3A^i^	3.177 (4)	H9A···C15B^xi^	3.3075
C13B···C14A^i^	3.296 (4)	H9A···H4A^xi^	2.8826
C13B···C7B^v^	3.437 (4)	H9A···H8B^xi^	3.4545
C13B···C8B^v^	3.560 (4)	H9A···H9B^xi^	2.4573
C15B···O1A^ix^	3.517 (4)	H1B···F1B^iii^	2.7751
C15B···O2A^ix^	3.347 (4)	H1B···O3B^iv^	2.4828
C15B···O3B^v^	3.526 (4)	H1B···C3B^iii^	3.5481
C15B···C13A^ix^	3.578 (4)	H1B···H2B^iv^	2.8982
C8B···O3A^i^	3.454 (4)	H1B···H8B^iii^	3.3691
C8B···C13B^v^	3.560 (4)	H2B···F1B^ix^	2.5957
C8B···C8B^v^	3.491 (4)	H2B···C9A^ix^	3.2766
F1A···H1A	2.4909	H2B···C2B^v^	3.5280
F1A···H7A	2.4029	H2B···C2B^ix^	3.4978
F1A···H9A	2.6103	H2B···C3B^v^	3.3883
F1B···H1B	2.4933	H2B···C3B^ix^	3.4818
F1B···H8B	2.3248	H2B···H3A^ix^	2.9605
F1B···H9B	2.7313	H2B···H1B^ix^	2.8982
O1A···H2A	2.4865	H6B···O3B^iv^	2.4851
O2A···H3A	2.5592	H6B···C1A	3.5681
O3A···H2A	2.4215	H6B···C7A	3.5890
O3A···H7A	3.0157	H6B···H8B^iii^	2.8843
O3A···H8A	2.4418	H5B···O2B^vi^	2.6011
O3A···H9A	2.9370	H5B···C11B^x^	3.2354
O1B···H2B	2.4889	H5B···C10B^x^	3.0902
O2B···H3B	2.5683	H5B···H5B^x^	3.2248
O3B···H2B	2.4589	H5B···H4B^x^	2.9805
O3B···H7B	2.4465	H5B···H3B^vi^	2.7987
O3B···H8B	2.9877	H4B···F1A	3.2409
O3B···H9B	2.9789	H4B···O2B^vi^	2.7742
C1A···H2A	3.2801	H4B···C3A	3.4446
C1A···H6A	2.7070	H4B···C11B^x^	3.3387
C2A···H6A	2.7263	H4B···C15B^xi^	2.9956
C3A···H2A	3.2398	H4B···H5B^x^	2.9805
C3A···H7A	2.9637	H4B···H7B^xi^	2.4979
C3A···H9A	3.1414	H4B···H8B^xi^	2.8437
C4A···H1A	3.2863	H4B···H9B^xi^	3.1786
C4A···H7A	2.7925	H3B···C5A^i^	3.4070
C4A···H8A	3.4078	H3B···H2A^i^	3.1616
C4A···H9A	2.8948	H3B···H5B^i^	2.7987
C6A···H1A	3.2511	H3B···H3B^v^	3.5899
C7A···H1A	2.7258	H3B···H7B^xi^	2.7783
C7A···H3A	3.2839	H7B···O1A^ix^	3.2290
C7A···H5A	3.2698	H7B···C13A^ix^	3.5973
C8A···H4A	3.2650	H7B···C10B^xii^	3.1932
C8A···H6A	3.2750	H7B···C9B^xii^	3.3247
C9A···H5A	3.2564	H7B···H4B^xii^	2.4979
C10A···H6A	3.2716	H7B···H3B^xii^	2.7783
C11A···H3A	3.2606	H8B···O3B^v^	2.8952
C13A···H3A	2.6106	H8B···C14B^v^	3.5242
C14A···H2A	2.6100	H8B···C12B^iii^	3.5381
C1B···H2B	3.2806	H8B···H9A^xii^	3.4545
C1B···H6B	2.7023	H8B···H1B^iii^	3.3691
C2B···H6B	2.7078	H8B···H6B^iii^	2.8843
C3B···H2B	3.2438	H8B···H4B^xii^	2.8437
C3B···H8B	2.9691	H9B···F1A^xii^	3.4807
C3B···H9B	3.1384	H9B···O1A^ix^	3.1562
C4B···H1B	3.2882	H9B···O2A^ix^	2.7803
C4B···H7B	3.4063	H9B···C13A^ix^	3.2166
C4B···H8B	2.8340	H9B···C15A^xii^	3.3534
C4B···H9B	2.8334	H9B···H4A	3.0698
C6B···H1B	3.2603	H9B···H7A^xii^	3.5311
C14B···H2B	2.6109	H9B···H9A^xii^	2.4573
C7B···H1B	2.7190	H9B···H4B^xii^	3.1786
C7B···H5B	3.2652		
			
C6A—O1A—C13A	122.05 (14)	C1B—C7B—C8B	118.13 (16)
C6B—O1B—C13B	122.21 (15)	C12B—C7B—C8B	118.71 (18)
C2A—C1A—C6A	117.24 (16)	C7B—C12B—C11B	120.18 (18)
C2A—C1A—C7A	124.22 (16)	C12B—C11B—C10B	120.84 (19)
C6A—C1A—C7A	118.54 (15)	C11B—C10B—C9B	120.14 (19)
C1A—C2A—C3A	119.90 (17)	C10B—C9B—C8B	119.31 (18)
F1A—C3A—C2A	117.02 (16)	C7A—C12A—C11A	120.21 (17)
F1A—C3A—C4A	119.66 (16)	O1B—C13B—O2B	116.95 (17)
C2A—C3A—C4A	123.32 (17)	O1B—C13B—C8B	117.28 (16)
C3A—C4A—C5A	116.53 (16)	O2B—C13B—C8B	125.76 (18)
C3A—C4A—C14A	125.70 (16)	C7B—C8B—C9B	120.82 (17)
C5A—C4A—C14A	117.78 (16)	C7B—C8B—C13B	121.36 (17)
C4A—C5A—C6A	120.66 (17)	C9B—C8B—C13B	117.82 (17)
O1A—C6A—C1A	122.04 (16)	C1A—C2A—H1A	120.044
O1A—C6A—C5A	115.62 (16)	C3A—C2A—H1A	120.053
C1A—C6A—C5A	122.34 (16)	C4A—C5A—H2A	119.672
C1A—C7A—C8A	118.28 (16)	C6A—C5A—H2A	119.667
C1A—C7A—C12A	123.01 (16)	C8A—C9A—H3A	119.881
C8A—C7A—C12A	118.70 (17)	C10A—C9A—H3A	119.871
C7A—C8A—C9A	120.54 (17)	C9A—C10A—H4A	120.235
C7A—C8A—C13A	120.99 (17)	C11A—C10A—H4A	120.235
C9A—C8A—C13A	118.47 (17)	C10A—C11A—H5A	119.615
C8A—C9A—C10A	120.25 (17)	C12A—C11A—H5A	119.611
C9A—C10A—C11A	119.53 (18)	C14A—C15A—H7A	109.473
C10A—C11A—C12A	120.77 (18)	C14A—C15A—H8A	109.469
O1A—C13A—O2A	116.56 (17)	C14A—C15A—H9A	109.471
O1A—C13A—C8A	118.09 (16)	H7A—C15A—H8A	109.473
O2A—C13A—C8A	125.34 (18)	H7A—C15A—H9A	109.470
O3A—C14A—C4A	118.58 (16)	H8A—C15A—H9A	109.470
O3A—C14A—C15A	120.17 (16)	C1B—C2B—H1B	120.248
C4A—C14A—C15A	121.25 (16)	C3B—C2B—H1B	120.241
C2B—C1B—C6B	117.54 (17)	C4B—C5B—H2B	119.735
C2B—C1B—C7B	123.51 (17)	C6B—C5B—H2B	119.726
C6B—C1B—C7B	118.94 (17)	C7B—C12B—H6B	119.910
C1B—C2B—C3B	119.51 (17)	C11B—C12B—H6B	119.911
F1B—C3B—C2B	116.95 (16)	C12B—C11B—H5B	119.574
F1B—C3B—C4B	119.61 (17)	C10B—C11B—H5B	119.585
C2B—C3B—C4B	123.44 (17)	C11B—C10B—H4B	119.936
C3B—C4B—C5B	116.74 (17)	C9B—C10B—H4B	119.926
C3B—C4B—C14B	125.49 (17)	C10B—C9B—H3B	120.341
C5B—C4B—C14B	117.76 (17)	C8B—C9B—H3B	120.351
C4B—C5B—C6B	120.54 (17)	C7A—C12A—H6A	119.901
O1B—C6B—C1B	122.04 (17)	C11A—C12A—H6A	119.891
O1B—C6B—C5B	115.73 (16)	C14B—C15B—H7B	109.477
C1B—C6B—C5B	122.23 (17)	C14B—C15B—H8B	109.477
O3B—C14B—C4B	119.10 (17)	C14B—C15B—H9B	109.459
O3B—C14B—C15B	120.32 (18)	H7B—C15B—H8B	109.474
C4B—C14B—C15B	120.57 (16)	H7B—C15B—H9B	109.468
C1B—C7B—C12B	123.16 (17)	H8B—C15B—H9B	109.472
			
C6A—O1A—C13A—O2A	−179.30 (15)	O3A—C14A—C15A—H9A	−112.0
C6A—O1A—C13A—C8A	1.3 (3)	C4A—C14A—C15A—H7A	−51.9
C13A—O1A—C6A—C1A	−0.3 (3)	C4A—C14A—C15A—H8A	−171.9
C13A—O1A—C6A—C5A	−179.78 (14)	C4A—C14A—C15A—H9A	68.1
C6B—O1B—C13B—O2B	−178.78 (14)	C2B—C1B—C6B—O1B	179.93 (14)
C6B—O1B—C13B—C8B	1.9 (3)	C2B—C1B—C6B—C5B	0.4 (3)
C13B—O1B—C6B—C1B	−0.0 (3)	C6B—C1B—C2B—C3B	0.4 (3)
C13B—O1B—C6B—C5B	179.59 (14)	C6B—C1B—C2B—H1B	−179.6
C2A—C1A—C6A—O1A	179.71 (15)	C2B—C1B—C7B—C12B	0.4 (3)
C2A—C1A—C6A—C5A	−0.9 (3)	C2B—C1B—C7B—C8B	179.50 (14)
C6A—C1A—C2A—C3A	0.5 (3)	C7B—C1B—C2B—C3B	−178.09 (14)
C6A—C1A—C2A—H1A	−179.5	C7B—C1B—C2B—H1B	1.9
C2A—C1A—C7A—C8A	−179.67 (14)	C6B—C1B—C7B—C12B	−178.07 (14)
C2A—C1A—C7A—C12A	1.0 (3)	C6B—C1B—C7B—C8B	1.0 (2)
C7A—C1A—C2A—C3A	−178.92 (14)	C7B—C1B—C6B—O1B	−1.5 (3)
C7A—C1A—C2A—H1A	1.1	C7B—C1B—C6B—C5B	178.91 (13)
C6A—C1A—C7A—C8A	0.9 (3)	C1B—C2B—C3B—F1B	179.49 (14)
C6A—C1A—C7A—C12A	−178.41 (14)	C1B—C2B—C3B—C4B	−0.8 (3)
C7A—C1A—C6A—O1A	−0.8 (3)	H1B—C2B—C3B—F1B	−0.5
C7A—C1A—C6A—C5A	178.61 (14)	H1B—C2B—C3B—C4B	179.2
C1A—C2A—C3A—F1A	−180.00 (15)	F1B—C3B—C4B—C5B	−179.91 (13)
C1A—C2A—C3A—C4A	0.2 (3)	F1B—C3B—C4B—C14B	1.6 (3)
H1A—C2A—C3A—F1A	0.0	C2B—C3B—C4B—C5B	0.3 (3)
H1A—C2A—C3A—C4A	−179.8	C2B—C3B—C4B—C14B	−178.12 (15)
F1A—C3A—C4A—C5A	179.66 (14)	C3B—C4B—C5B—C6B	0.4 (3)
F1A—C3A—C4A—C14A	−0.4 (3)	C3B—C4B—C5B—H2B	−179.6
C2A—C3A—C4A—C5A	−0.5 (3)	C3B—C4B—C14B—O3B	164.33 (17)
C2A—C3A—C4A—C14A	179.39 (16)	C3B—C4B—C14B—C15B	−16.6 (3)
C3A—C4A—C5A—C6A	0.2 (3)	C5B—C4B—C14B—O3B	−14.1 (3)
C3A—C4A—C5A—H2A	−179.8	C5B—C4B—C14B—C15B	164.96 (15)
C3A—C4A—C14A—O3A	178.88 (17)	C14B—C4B—C5B—C6B	179.01 (14)
C3A—C4A—C14A—C15A	−1.3 (3)	C14B—C4B—C5B—H2B	−1.0
C5A—C4A—C14A—O3A	−1.2 (3)	C4B—C5B—C6B—O1B	179.62 (15)
C5A—C4A—C14A—C15A	178.62 (15)	C4B—C5B—C6B—C1B	−0.8 (3)
C14A—C4A—C5A—C6A	−179.75 (15)	H2B—C5B—C6B—O1B	−0.4
C14A—C4A—C5A—H2A	0.3	H2B—C5B—C6B—C1B	179.2
C4A—C5A—C6A—O1A	179.99 (15)	O3B—C14B—C15B—H7B	−0.9
C4A—C5A—C6A—C1A	0.5 (3)	O3B—C14B—C15B—H8B	−120.9
H2A—C5A—C6A—O1A	−0.0	O3B—C14B—C15B—H9B	119.1
H2A—C5A—C6A—C1A	−179.5	C4B—C14B—C15B—H7B	−180.0
C1A—C7A—C8A—C9A	−179.62 (13)	C4B—C14B—C15B—H8B	60.0
C1A—C7A—C8A—C13A	0.1 (3)	C4B—C14B—C15B—H9B	−60.0
C1A—C7A—C12A—C11A	179.45 (13)	C1B—C7B—C12B—C11B	178.81 (14)
C1A—C7A—C12A—H6A	−0.6	C1B—C7B—C12B—H6B	−1.2
C8A—C7A—C12A—C11A	0.1 (3)	C1B—C7B—C8B—C9B	−179.09 (13)
C8A—C7A—C12A—H6A	−179.9	C1B—C7B—C8B—C13B	0.8 (3)
C12A—C7A—C8A—C9A	−0.3 (3)	C12B—C7B—C8B—C9B	0.1 (3)
C12A—C7A—C8A—C13A	179.41 (14)	C12B—C7B—C8B—C13B	179.99 (14)
C7A—C8A—C9A—C10A	0.1 (3)	C8B—C7B—C12B—C11B	−0.3 (3)
C7A—C8A—C9A—H3A	−179.9	C8B—C7B—C12B—H6B	179.7
C7A—C8A—C13A—O1A	−1.2 (3)	C7B—C12B—C11B—C10B	0.2 (3)
C7A—C8A—C13A—O2A	179.49 (16)	C7B—C12B—C11B—H5B	−179.8
C9A—C8A—C13A—O1A	178.53 (15)	H6B—C12B—C11B—C10B	−179.8
C9A—C8A—C13A—O2A	−0.8 (3)	H6B—C12B—C11B—H5B	0.2
C13A—C8A—C9A—C10A	−179.54 (15)	C12B—C11B—C10B—C9B	0.1 (3)
C13A—C8A—C9A—H3A	0.5	C12B—C11B—C10B—H4B	−179.9
C8A—C9A—C10A—C11A	0.1 (3)	H5B—C11B—C10B—C9B	−179.9
C8A—C9A—C10A—H4A	−179.9	H5B—C11B—C10B—H4B	0.1
H3A—C9A—C10A—C11A	−179.9	C11B—C10B—C9B—C8B	−0.4 (3)
H3A—C9A—C10A—H4A	0.1	C11B—C10B—C9B—H3B	179.6
C9A—C10A—C11A—C12A	−0.2 (3)	H4B—C10B—C9B—C8B	179.6
C9A—C10A—C11A—H5A	179.7	H4B—C10B—C9B—H3B	−0.4
H4A—C10A—C11A—C12A	179.8	C10B—C9B—C8B—C7B	0.3 (3)
H4A—C10A—C11A—H5A	−0.2	C10B—C9B—C8B—C13B	−179.66 (15)
C10A—C11A—C12A—C7A	0.1 (3)	H3B—C9B—C8B—C7B	−179.7
C10A—C11A—C12A—H6A	−179.9	H3B—C9B—C8B—C13B	0.4
H5A—C11A—C12A—C7A	−179.9	O1B—C13B—C8B—C7B	−2.3 (3)
H5A—C11A—C12A—H6A	0.1	O1B—C13B—C8B—C9B	177.64 (13)
O3A—C14A—C15A—H7A	128.0	O2B—C13B—C8B—C7B	178.43 (16)
O3A—C14A—C15A—H8A	8.0	O2B—C13B—C8B—C9B	−1.6 (3)

Symmetry codes: (i) *x*, −*y*+2, *z*+1/2; (ii) −*x*+1/2, −*y*+3/2, −*z*; (iii) −*x*+1, −*y*+1, −*z*; (iv) *x*, −*y*+1, *z*−1/2; (v) −*x*+1, *y*, −*z*+1/2; (vi) *x*, −*y*+2, *z*−1/2; (vii) −*x*+1/2, *y*−1/2, −*z*−1/2; (viii) −*x*+1/2, *y*+1/2, −*z*−1/2; (ix) *x*, −*y*+1, *z*+1/2; (x) −*x*+1, −*y*+2, −*z*; (xi) *x*, *y*+1, *z*; (xii) *x*, *y*−1, *z*; (xiii) −*x*+1/2, −*y*+5/2, −*z*.
